# The role of interleukin 17-mediated immune response in Chagas disease: High level is correlated with better left ventricular function

**DOI:** 10.1371/journal.pone.0172833

**Published:** 2017-03-09

**Authors:** Giovane R. Sousa, Juliana A. S. Gomes, Marcos Paulo S. Damasio, Maria Carmo P. Nunes, Henrique S. Costa, Nayara I. Medeiros, Rafaelle C. G. Fares, Ana Thereza Chaves, Rodrigo Corrêa-Oliveira, Manoel Otávio C. Rocha

**Affiliations:** 1 Postgraduate Course of Infectious Diseases and Tropical Medicine, School of Medicine, Universidade Federal de Minas Gerais, Belo Horizonte, MG, Brazil; 2 Laboratory of Cell-Cell Interactions, Department of Morphology, Institute of Biological Sciences, Universidade Federal de Minas Gerais, Belo Horizonte, MG, Brazil; 3 Laboratory of Cellular and Molecular Immunology, Centro de Pesquisas René Rachou, FIOCRUZ, Belo Horizonte, MG, Brazil; 4 National Institutes of Science and Technology Tropical Diseases (INCT-DT), Belo Horizonte, MG, Brazil; Rutgers University, UNITED STATES

## Abstract

Interleukin 17A (IL-17A) has been associated with protective rather than pathogenic response in Chagas disease (ChD). However, it is not established whether or not IL-17A-mediated immune response is correlated with patient’s left ventricular (LV) function in ChD. To address this question we have gathered cardiac functional parameters from ChD patients and analysed the possible relationship between their plasma IL-17A levels and LV function. Plasma IL-17A levels were measured by BD Cytometric Bead Array (CBA) in 240 patients with positive specific serology for *Trypanosoma cruzi (T*. *cruzi)* grouped as indeterminate (IND) and Chagas cardiomyopathy (CARD) forms. The levels of IL-17A in ChD patients were compared with 32 healthy individuals, mean age of 39 years, 50% male, that were also included as a control group (non-infected [NI]). The overall mean age of ChD patients was 46 years and 52% were male. The IND group included 95 asymptomatic patients, with ages ranging from 27 to 69 years (mean of 43 years), and 42.1% of them were male. The CARD group included 145 patients, which 58.6% were male, with ages ranging from 23 to 67 years (mean of 49). The IND group presented substantially higher levels of IL-17A, median of 26.16 (3.66–48.33) as compared to both the CARD group, median of 13.89 (3.87–34.54) (*P* <0.0001), and the NI group, median of 10.78 (6.23–22.26) (*P* <0.0001). The data analysis demonstrated that the IND group comprises a significantly greater proportion (*P* <0.001) of high IL-17A producers (52.6%, 50 of 95 subjects) than do the other groups. A significant direct correlation was verified between IL-17A levels and cardiac function expressed by LV ejection fraction (LVEF), LV diastolic diameter (LVDd), and body surface area (BSA)-indexed LVDd as well as ratio of the early diastolic transmitral flow velocity to early diastolic mitral annular velocity (E/e’) in both groups. We demonstrated that plasma IL-17A levels has an accurate sensitivity and specificity to predict heart failure in serology-positive patients and might be a useful parameter to distinguish patients with or without cardiac impairment. This study indicates a consistent relationship between high expression of IL-17A and better LV in human chronic ChD. Our data raise the possibility that IL-17A plays an important immunomodulatory role in the chronic phase of ChD and might be involved in protection against myocardial damage.

## Introduction

The immunopathogenesis of Chagas disease (ChD) is complex and not yet fully understood [1]. Interleukin 17A (IL-17A) is a proinflammatory cytokine that contributes to host protection against a range of infectious pathogens by inducing the recruitment of neutrophils and secretion of inflammatory mediators [2, 3]. Prior literature has suggested an important role for IL-17A in the resolution of infection with the protozoan parasite *T*. *cruzi* [4–6]. Moreover, preliminary data both from experimental [7] and human *T*. *cruzi* infection studies [8–10] have indicate that this cytokine is associated with protective rather than pathogenic responses.

Studies on IL-17A in human ChD are limited and have usually employed small sample size [8–11]. In addition, only in one study, correlation analysis was performed between this cytokine and parameters of cardiac function such as LVEF and LVDD [10]. Confliting evidence exists regarding the role of IL-17A-mediated immune response in human ChD. Such investigations require larger sample sets of cases and controls. Given these findings, this study was designed to evaluate the plasma IL-17A levels in patients with indeterminate and severe cardiac forms of ChD and to assess whether or not IL-17A-mediated immune response would correlate with patients’ LV function.

These clinical forms were selected to this study since they represent extremes of the wide clinical spectrum of ChD. The indeterminate form of ChD is defined by the presence of *T*. *cruzi* infection, absence of symptoms, and of electrocardiographic and radiologic abnormalities [12]. In contrast, Chagas cardiomyopathy is characterized by chronic myocarditis that involves all cardiac chambers and damage to the conduction system [13]. This most severe form of ChD manifests as three frequent syndromes that can co-occur in an individual patient: heart failure, cardiac arrhythmia, and thromboembolism [14]. These patients are of special interest for studies involving the natural development of the disease and the possible determinants of clinical outcome. The identification of non-invasive markers related to morbidity and prognosis may allow the recognition of subgroups of patients with potential to progress toward the severe forms of ChD, which should require special attention and specific therapeutic approaches in order to prevent or delay the unfavorable outcome of these patients. In this study, we gathered cardiac functional parameters from ChD patients, aged 23 to 69 years and analysed the possible relationship between their plasma IL-17A levels and LV function.

## Materials and methods

### Study population

This study comprises 240 patients in the chronic phase of ChD from endemic areas within the state of Minas Gerais, Brazil, who were identified and selected at the Referral Outpatient Center for Chagas Disease at the Clinical Hospital of the Universidade Federal de Minas Gerais (UFMG), Brazil. All patients were tested positive for *T*. *cruzi* and fulfilled eligibility criteria. Positive specific serology for *T*. *cruzi* was determined by two or more tests (indirect immunofluorescence, enzyme-linked immunosorbent assay [ELISA], or indirect hemagglutination). The study protocol was approved by the Research Ethics Committee of the Universidade Federal de Minas Gerais (protocol COEP-ETHIC 502/11) and Centro de Pesquisas René Rachou (protocol CEPSH-CPqRR 15/2011) and all participants provided written informed consent.

Inclusion criteria were the diagnosis of IND defined as asymptomatic individuals, in sinus rhythm, with no significant alterations in electrocardiography, chest radiograph, and echocardiogram, and diagnosis of CARD characterized by the echocardiographic finding of a dilated left ventricle with impaired ventricular systolic function. LVEF, LVDd, and E/e’ ratio were used as variables of LV function, where LVEF <55% and LVDd/BSA ≥31mm were used to define CARD patients [12]. Healthy individuals from a non-endemic area for ChD and showing negative serological tests for the infection were included as a control group, NI. None of the patients were undergoing etiological treatment nor had been previously treated for *T*. *cruzi* infection. Individuals with any other chronic inflammatory disease, thyroid dysfunction, valvular heart disease, rheumatic or ischemic heart disease, coronary artery disease, systemic arterial hypertension, chronic obstructive pulmonary disease, hydroelectrolytic disorders, kidney disease, diabetes mellitus, alcoholism, pregnancy defined by laboratorial criteria, and other cardiomyopathies or infectious diseases were excluded from this study. Eligible and consenting subjects were enrolled on the study.

### Ecochardiogram evaluation

A standard transthoracic two-dimensional (2D) echocardiogram was performe using commercially available equipment (iE33, Philips Medical Systems, Andover, MA) according to recommendations of the American Society of Echocardiography [15]. LV ejection fraction was calculated according to the modified Simpson’s rule.LV diastolic function was assessed by pulsed Doppler of the mitral inflow and by tissue Doppler imaging measurements, obtained at the medial and lateral border of the mitral annulus in the apical 4-chamber view [15]. Systolic tissue Doppler velocity (Sʹ), and early (eʹ) and late (Aʹ) diastolic tissue velocities were acquired, and the ratio of the mitral E velocity to the mean eʹ’ was calculated (E/eʹ).

### Multiplexed bead-based immunoassay

A BD CBA Human Th1/Th2/Th17 cytokine Kit (Catalog No. 560484; Becton Dickinson Biosciences, San Jose, CA, USA) was used to quantify IL-17A concentration in plasma, following manufacturer instructions. The data were acquired in a BD FACSCalibur flow cytometry system (BD Biosciences, San Jose, CA, USA) and analyses performed using BD FCAP Array v3.0 software (BD Biosciences, San Jose, CA, USA). The standard curve was determined using a five-parameter logistic (5-PL) equation. The results were based on standard concentration curve and expressed as picogram per milliliter (pg/mL).

### Statistical analysis

Statistical comparative analyses were performed in groups of two between the NI, IND, and CARD groups, using the non-parametric Kruskal-Wallis test and Mann-Whitney U test, together with the Bonferroni correction (significance level, 0.05/3 = 0.0167) [16]. Categorical data were presented as numbers and percentages, and continuous data were expressed as mean ± standard deviation (SD) or median and interquartile range (25%-75%), as appropriate, based on the prior Shapiro-Wilk normality test.

IBM SPSS Statistics 20.0 software (SPSS Inc., Chicago, Illinois) was used for all analyses. Graphs were prepared using GraphPad Prism v7.0 software (San Diego, CA, USA). Results were nominally significant at two-sided *P* < 0.05.

## Results

### Characteristics of the study population

The major clinical, eletrocardiographic, and echocardiographic characteristics of the study population are reported in Table 1. A total of 272 subjects were included in the study. Overall, this cohort consists of middle-aged (mean age, 46 years) subjects with 52% of males. The mean age of ChD patients was 46±9.9 years, and 52% were male. The IND group included 95 asymptomatic patients, with ages ranging from 27 to 69 years (mean of 43±9). The CARD group included 145 patients, with ages ranging from 23 to 67 years (mean of 49±10). The CARD group had significantly greater proportion of males than other groups and most of the CARD patients were in NYHA functional class I or II (84.1%). Moreover, 32 healthy individuals, 50% males, ranging from 27 to 58 years of age (mean of 39±10), were also included as the NI group. As expected, the mean QRS duration in the IND group was similar to that observed in the NI group, 83.2±2.6 ms and 81.4±2.9 ms, respectively. QRS duration was increased in all CARD patients with mean of 124.6±26 ms. Corrected QT duration was within normal limits and was similar in the three groups (Table 1). Right bundle-branch block (RBBB) was the most frequent electrocardiogram (ECG) abnormality, observed in 52 of 145 (36%) of CARD patients. Of the CARD patients with RBBB, 41 patients had associated left anterior fascicular block (LAFB) and 1 had left posterior fascicular block (LPFB). Left bundle branch block (LBBB) and first-degree atrioventricular blocks (AVB) were detected, respectively, in 3 (2%) and 13 (9%) of 145 CARD patients. Atrial fibrillation was present in 6 of 145 (4%) of CARD patients and all these patients were taking anticoagulants. Neither NI subjects nor IND patients had RBBB (with or without associated LAFB), LBBB, LPFB, or other significant ECG abnormality that would motivate the exclusion from this study.

**Table 1:** Clinical, eletrocardiographic, and echocardiographic characteristics of the study population.

Characteristic	Healthy control (*n* = 32)	Indeterminate form (*n* = 95)	Chagas cardiomyopathy (*n* = 145)	*P*-value*
Age, yr	39±10	43±9	49±10	0.0012
Male sex, *n* (%)	16 (50)	40 (42.1)	85 (58.6)	0.0026
Body-surface area, m^2^	1.8 (1.7, 1.9)	1.8 (1.7, 1.8)	1.6 (1.6, 1.7)	0.0189
Heart rate, beats/min	69 (63, 76)	68 (62, 76)	63 (60, 74)	0.0203
Blood pressure, mm Hg				
• Systolic	116±8	119±10	107±12	<0.0001
• Diastolic	79±7	82±7	71±10	<0.0001
Eletrocardiographic				
• QRS duration, ms	81.4±2.9	83.2±2.6	124.6±26	0.0037
• Corrected QT interval, ms	451±32	453±35	453±41	0.4565
Echocardiographic				
• LV ejection fraction, %	68 (66, 70)	66 (63, 69)	38 (29, 47)	<0.0001
• LV end-diastolic diameter, mm	45 (43, 47)	48 (45, 50)	63 (59, 68)	<0.0001
• LV end-diastolic diameter/BSA, mm/m^2^	25 (23, 26)	27 (25, 28)	37 (34, 41)	<0.0001
• E/e’ ratio	7.3 (6.2, 10.1)	8.2 (6.3, 10.1)	10.3 (7.5, 14.2)	<0.0001

Values are expressed as mean and standard deviation (mean±SD), median (interquartile range), or absolute number and percentage. E/e’ratio, ratio of the early diastolic transmitral flow velocity to early diastolic mitral annular velocity; LAFB, left anterior fascicular block; LV, left ventricular; RBBB, right bundle branch block; RBBB, left bundle branch block.

*P-value< 0.05 (two-sided) for comparison between the groups (Kruskal-Wallis test).

### Plasma IL-17A levels are associated with morbidity in Chagas disease

**Fig 1:**
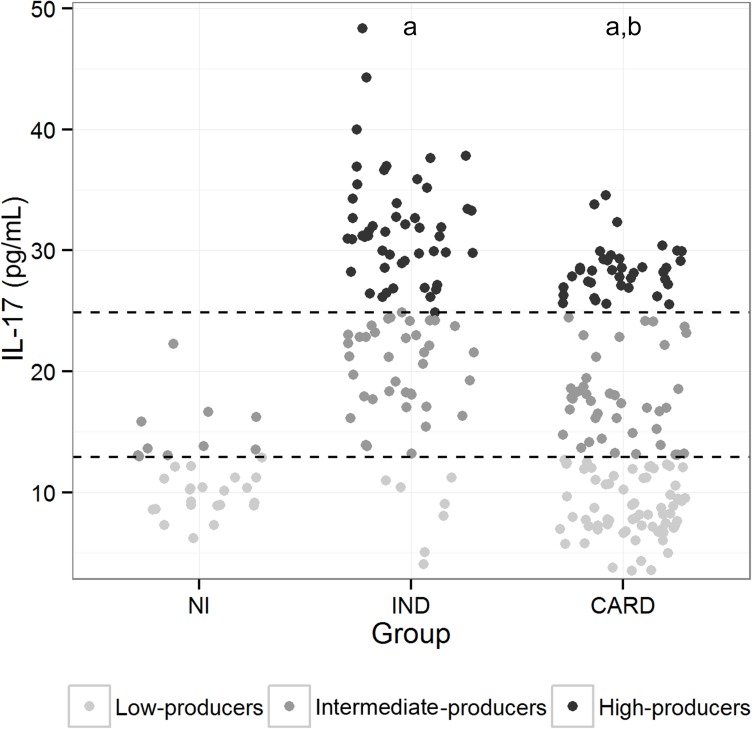
Analyses of plasma IL-17A levels in the study groups. Representative scatter plot graph of plasma IL-17A used to establish the cutoff to define low, intermediate, and high cytokine producers. Low IL-17A producers were defined by values of lower than the first tertile. Intermediate IL-17A producers were defined by values equal to or lower than the second tertile, while high IL-17A producers were defined by values higher than or equal to the second tertile [18]. The results were expressed by pg/mL. Results were considered significant with a *P*-value <0.05.

To determine whether the levels of IL-17A are associated with morbidity in ChD, the concentration of this cytokine was evaluated in the plasma from IND and CARD patients as well as healthy individuals. Differences among the three groups were observed for IL-17A (*P* <0.0001). The IND group presented substantially higher levels of IL-17A, median of 26.16 (3.66–48.33) as compared to the CARD group, median of 13.89 (3.87–34.54) (*P* <0.0001), and the NI group, median of 10.78 (6.23–22.26) (*P* <0.0001) (Fig 1). To establish the IL-17A cutoff that would permit the identification of patients with IND and CARD forms of ChD, an analysis was performed that allows a division of the individuals into three categories: low (3.66–12.69 pg/mL), intermediate (12.88–24.88 pg/mL), and high (24.89–48.33 pg/mL) IL-17A producers. To that end, these three groups were identified based on the detailed analysis of individual levels of IL-17A obtained for NI, IND, and CARD groups; a statistical method based in tertiles [17] was employed to determine the cut-off for IL-17A plasma levels, as shown in Fig 1. Data analysis demonstrated that the IND group comprises a significantly higher frequency of intermediate (40% 38/95) and high (52.63% 50/95) IL-17A producers than do the other groups (*P* <0.001) (Fig 1). In contrast, most of the CARD patients displayed values of IL-17A levels that are below the cutoff for low IL-17A producers, being significantly lower than IND patients (7.36%, 7/95) (*P* <0.001), while approximately 30.34% (44/145) and 26.89% (39/145) of CARD patients were identified as intermediate and high IL-17A producers, respectively (Fig 1). None of the individuals were identified as high IL-17A producers in the NI group (Fig 1).

### High IL-17A levels correlated with better left ventricular function in Chagas disease

**Fig 2:**
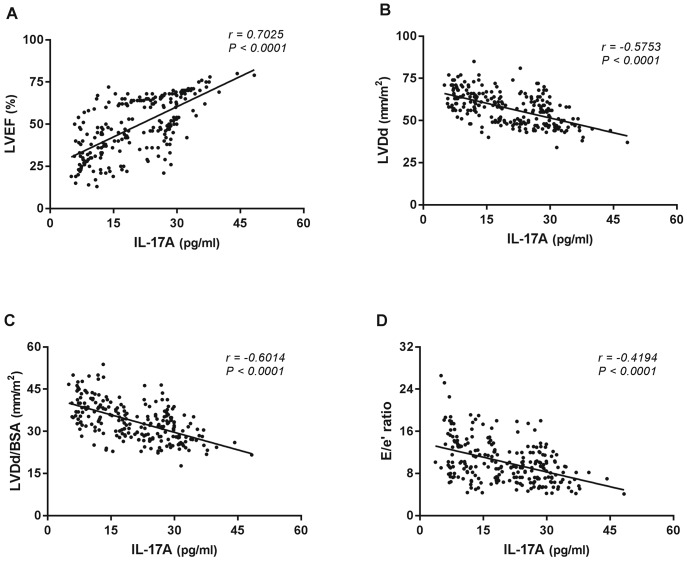
Correlation analysis between plasma IL-17A levels and echocardiographic parameters of left ventricular function in Chagas disease patients. Correlation analysis was performed between plasma IL-17A levels and variables of LV function (LVEF, LVDd, LVDd/BSA, and E/e’ ratio) in the ChD patients as a whole. The analysis was performed using the Spearman correlation coefficient, and results were considered significant when *P*-value <0.05. Significant differences are indicated in each graph together with the r value.

In order to investigate the existence of association between IL-17A levels and LV function in ChD, correlation analysis was performed between the plasma levels of IL-17A in either ChD patients and healthy non-chagasic individuals and variables of LV systolic and diastolic ventricular functions. Data analysis indicated a relationship between lower levels of IL-17A and worse cardiac function in ChD. Interestingly, a significant direct correlation was identified between IL-17A levels and LVEF in ChD patients as a whole (Fig 2A). In addition, a significant inverse correlations between IL-17A levels and either LVDd (Fig 2B), LVDd/BSA (Fig 2C) or E/e’ ratio (Fig 2D) could be observed. In contrast, no linear relationship could be found between IL-17A levels and variables of LV ventricular function in the NI group.

**Fig 3:**
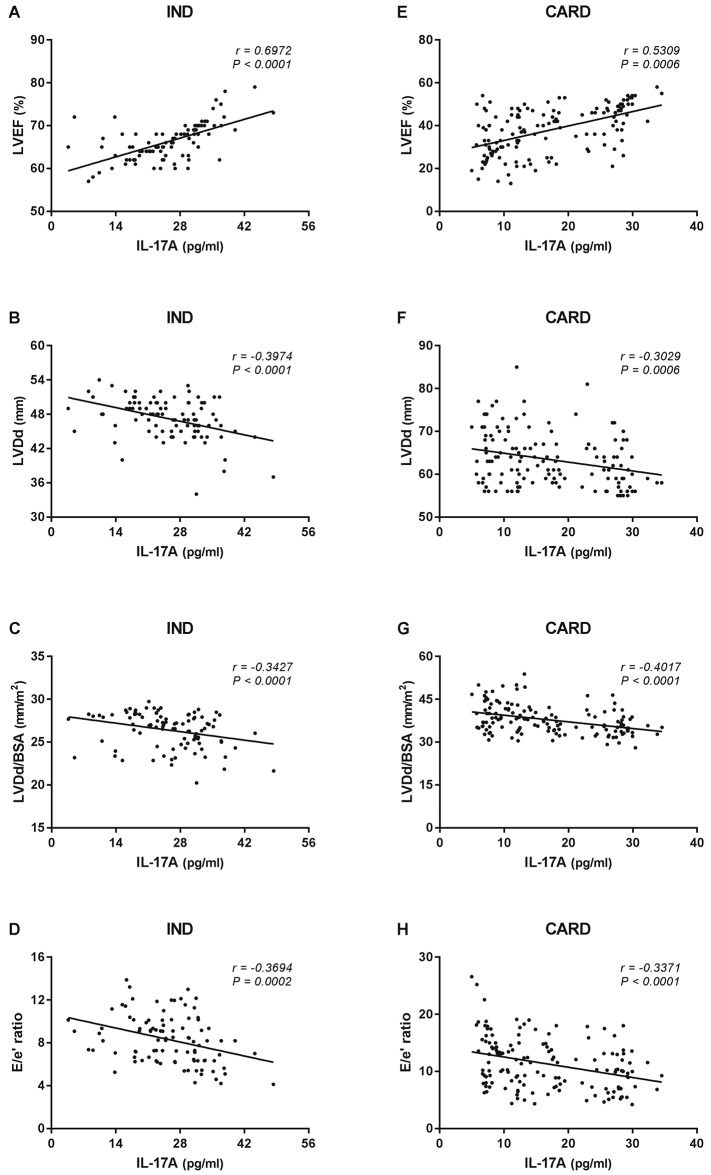
Correlation analysis between plasma IL-17A levels and echocardiographic parameters of left ventricular function according to the form of Chagas disease. Correlation analysis was performed between plasma IL-17A levels and parameters of LV function (LVEF, LVDd, LVDd/BSA, and E/e’ ratio) in the IND (n = 95, A, B, C, and D, respectively) and CARD (n = 145, E, F, G, and H, respectively) groups. The analysis was performed using the Spearman correlation coefficient, and results were considered significant when *P*-value <0.05. Significant differences are indicated in each graph together with the r value.

When the association between the levels of IL-17A and LVEF was investigated in the IND group, a significant direct correlation was observed (r = 0.6972, *P* <0.0001) (Fig 3A). In contrast, a significant inverse correlation could be found between IL-17A levels and both LVDd (r = -0.3974, *P* <0.0001) (Fig 3B) and LVDd/BSA (r = -0.3427, *P* <0.0001) in the same group (Fig 3C). Plasma IL-17A levels were also inversely correlated with E/e’ ratio in the IND group (r = -0.3694, *P* <0.0001) (Fig 3D). A significant direct correlation was identified when IL-17A levels were compared with LVEF in the CARD group (r = 0.5309, *P* <0.0001) (Fig 3E). Conversely, inverse correlation was observed between IL-17A levels and both LVDd (r = -0.3029, *P* = 0.0006) (Fig 3F) and LVDd/BSA (r = -0.4017, *P* = 0.0001) (Fig 3G) in the CARD group. Similar results could be found when comparing IL-17A levels and E/e’ ratio in the same group (r = -0.3371, *P* = 0.0001) (Fig 3H).

**Fig 4:**
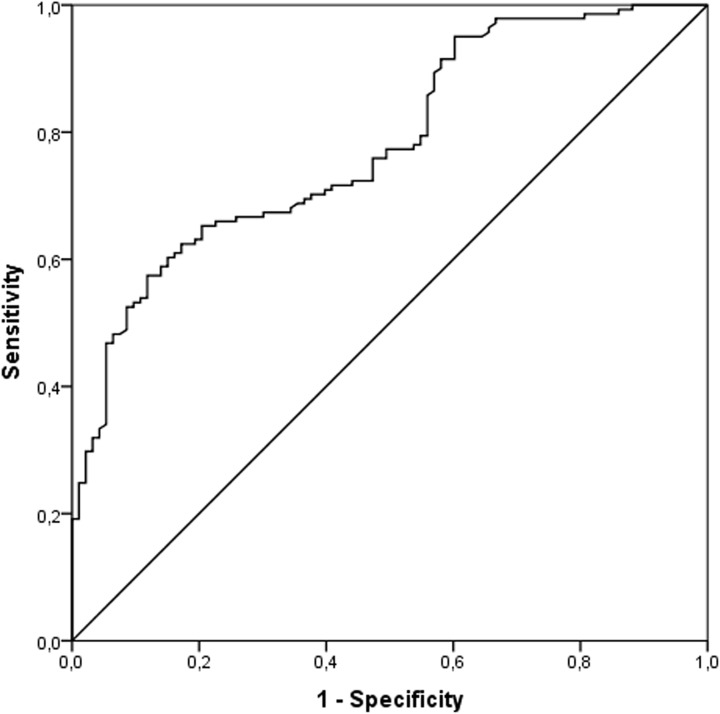
Receiver operating characteristics curve of sensitivity of heart failure prediction against specificity. A risk score was calculated on the basis of the plasma IL-17A levels in the IND and CARD groups by logistic regression, and the proportion of all cases that score greater than any given cutoff (sensitivity) against the proportion of the IND patients that would also exceed the same cutoff (specificity) was plotted. The slope of the tangent line at a cutpoint indicates the likelihood ratio for that value of the test and the diagonal line represents random prediction.

Although, the IL-17A levels correlated directly with better left ventricular function in chronic ChD, we sought to obtain additional evidence of the strength of association. Considering that in clinical workup the determination of plasma IL-17A levels would be achieved to downstream to Chagas serology, a Receiver Operating Characteristic (ROC) curve analysis was performed based on the IND and CARD groups. The contribution of IL-17A levels to discriminate diseased cases with heart failure (CARD) from infected patients without cardiac impairment (IND) is shown in the ROC curve (Fig 4). The area under the ROC curve (AUC) to predict HF (Fig 4) was 0.78 (95% CI: 0.722, 0.838; *P* <0.0001). The optimal threshold of IL-17A to predict CARD in serology-positive patients was less than 17.97 pg/mL based on accurate sensitivity and specificity of 61% and 84%, respectively.

## Discussion

The principal findings of this study were: (a) The plasma IL-17A levels were at least 2-fold higher in the IND group than those in the NI and CARD groups; (b) The IND group presented a significantly greater proportion of high IL-17A producers than do the other groups; and (c) high plasma levels of IL-17A is associated with better LV function in ChD.

There was also a greater proportion of males in the CARD group; however, an association between male sex and risk progression to chronic Chagas cardiomyopathy (CCC) or death in patients with ChD has previously been reported [18–20]. A previous study in a endemic area including asymptomatic *T*. *cruzi*–seropositive blood donors demonstrated that male sex was associated with the onset of cardiomyopathy [21]. Therefore, in the present study the greater proportion of male in CARD group was expected.

The present study included quantitative cardiac functional parameters to assess the LV function of the study population. In CCC patients, impairment of LV filling and systolic dysfunction usually coexist [22–24] and are consistent predictors of functional capacity [25, 26] and mortality [27] in this severe form of ChD. A previous study showed that patients with mild or moderate systolic dysfunction, the E/e’ ratio (an important marker of LV filling pressure) of more than 15 was powerful in predicting prognosis in patients with Chagas cardiomyopathy [28].

Higher levels of IL-17A in the IND group compared to the NI group might be explained by a possible effect of *T*. *cruzi* infection inducing immune response even in these asymptomatic patients. This result is in agreement with findings from previous studies [8–10]. A significant frequency of IND patients presented high levels of IL-17A, whereas most of the CARD patients expressed low levels of this cytokine. Although, low and high producers of IL-17A were observed in both groups. The importance of this differential expression throughout the chronic phase of ChD and to the disease outcome remains unknown. A possible explanation, for the broad distribution range of IL-17A levels produced by CARD patients, could be the difference in the expression of immune response determined by different host’s genetic patterns. Furthermore, it could be hypothesized that lower IL-17A producers in the CARD group would develop an earlier or worse LV function. On the other hand, higher IL-17A producers in the same group probably would display later and less intense cardiac morbidity. In contrast, lower IL-17A producers in the IND group would be those likely to develop myocardial damage, whereas those that show higher levels of this cytokine would tend to present a later cardiac involvement or remain asymptomatic. In this context, it is seems reasonable to consider that intermediate IL-17A producers might present an expression profile resulting of balanced immune response controlled by regulatory mechanisms such as Foxp3+CD25(high)CD4+ regulatory T cells previously demonstrated by our group [8]. Nevertheless, the disease progression and clinical future of those patients has not been established yet and probably involve multiple factors.

We also addressed the question of whether or not IL-17A-mediated immune response is correlated with patients’ LV. Notably, a significant correlation was observed between the plasma IL-17A levels in either IND or CARD groups and cardiac involvement expressed by variables of systolic (LVEF) and diastolic (LVDd, LVDd/BSA and E/e’ ratio) ventricular function. Overall, these data showed that high plasma levels of IL-17A are associated with better LV function. Thus, it is tempting to speculate that IL-17A might be linked to a protective role in the chronic phase of ChD. While our data indicate a consistent relationship between high IL-17A levels and better cardiac function in human ChD, this and other previous studies in patients with ChD were cross-sectional and could not establish causality between heart damage protection and IL-17A expression. In-depth basic and translational investigations aimed at understanding the molecular immunology and the pathophysiology are needed to elucidate the mechanisms underlying the IL-17A expression in long-term chronic ChD. Our longitudinal study cohort in progress provide an opportunity to investigate the influence of IL-17A expression on disease progression and clinical outcome since multiple samples are available from the same patients and healthy control subjects without ChD.

The findings from prior studies have suggested a possible protective role of IL-17A in experimental infection models [4] and in human ChD [10]. In order to better understand the role of IL-17A in *T*. *cruzi* infection, IL-17A^−/−^ mice and anti-IL-17A treatment were used as strategy [4, 5]. In this study, it was verified earlier mortality of IL-17A^−/−^ mice when compared to mice controls treated with anti-IL-17A. These studies suggest that IL-17A would be relevant for the balance of immune response and important for parasite control during the chronic phase of the disease [1]. However, a recent study investigating the role of IL-17A in non-chagasic inflammatory dilalated cardiomyopathy–an important cause of noncongenital heart failure in individuals under the age of 40, has reported that IL-17A/IL-17RA signaling pathway is required for the development of the disease [29]. It was demonstrated that IL-17RA^−/−^ develop limited cardiac enlargement and fibrosis. They further showed that IL-17A was associated with induction of chemokine production by fibroblasts, which attracts effector cells to the myocardium and increases inflammation [29]. In ChD context, increased expression of IL-17A mRNA was found in the myocardial inflammatory infiltrate of CCC patients [30]. In this cross-sectional study, the expression of IL-17A was evaluated in myocardial samples from 14 end-stage heart failure CCC patients, 8 end-stage heart failure patients with noninflammatory cardiomyopathies (5 patients with idiopathic dilated cardiomyopathy and 3 patients with ischemic cardiomyopathy, all seronegative for *T*. *cruzi*), and 6 nonfailing donor hearts. However, patients with indeterminate form of ChD–important subjects to study the immune response and its association with disease morbidity and mortality in chronic ChD were not included in the investigation.

In the present study we have examined the expression of IL-17A in plasma samples from a large set of IND and CARD patients, and healthy controls. We demonstrated that plasma IL-17A levels has an accurate sensitivity and specificity to predict heart failure in serology-positive patients and might be a useful parameter to distinguish patients with or without cardiac impairment, as CARD and IND patients, respectively. Our findings indicate a consistent relationship between high expression of IL-17A and better LV in human chronic ChD. Nonetheless, observations on human plasma samples may not reflect which would be observed in myocardial LV free wall heart specimens from IND and CARD patients.

IL-17A has been described as a cytokine secreted by T helper 17 cells (Th17). Moreover, other cells were recognized as IL-17A sources, such as monocytes, neutrophils, and natural killer T (NKT) cells [2, 3, 31]. Bermejo *et al*. showed that B cells are the principal source of IL-17A after infection with the extracellular parasite *T*. *cruzi* and that the B cells produce IL-17A independently of RORγT, which is considered the main transcription factor involved with Th17 differentiation [6]. Fibroblasts, macrophages, neutrophils, B and T lymphocytes are considered target cells of this cytokine [3]. Once IL-17A binds to its receptor, it induces the secretion of inflammatory cytokines (TNF, IL-1 and IL-6) and chemokines (CXCL1, CXCL5, IL-8, CCL2, and CCL7). Like many cytokines, lL-17A may play a role in tumor development, encephalomyelitis, arthritis, systemic lupus erythematosus, inflammatory bowel disease, sepsis, and allergic diseases [31, 32]. In contrast to pathogenic roles of IL-17A in autoimmune and chronic inflammatory diseases, this cytokine promotes host defense against pathogens at epithelial and mucosal tissues which include the skin, lung, and instetine [2, 3, 31].

IL-17A induces the expression of several proinflammatory chemokines and cytokines through activation of NF-κB, MAPKs and C/EBPs cascades [32–34]. Furthermore, other studies have indicated that IL-17A can activate JAK–PI3K and JAK–STAT pathways [35, 36]. However, how IL-17A contributes to the activation of these pathways is unclear. Hence, further investigations will be necessary to identify the signaling pathway and the effects of IL-17A in distinct cell populations such as CD8+ T cells, macrophages, and NKT cells. It is also important to evaluate the signaling induced by IL-17A considering the presence of antigens and different gene polymorphisms.

In this study, we included a large and homogenous cohort of ChD patients who have not been previously treated for *T*. *cruzi* infection and performed a strategic analysis to establish an appropriate determination of cutoff edge that allows the division of IL-17A levels in three categories [37]. This method of analysis dichotomize low and high producers of IL-17A –extremes of expression, and express better a biological phenomenon that presents a wide dispersion.

In conclusion, our data raise the possibility that IL-17A plays an important immunomodulatory role in the chronic phase of ChD and might be involved in protection against myocardial damage. However, there are still many open questions pertaining the role of IL-17A in *T*. *cruzi* infection [38] and further studies will be necessary to address them. To elucidate the role of IL-17A in the development or control of chronic Chagas cardiomyopathy, we are now also investigating whether lower levels of IL-17A are related to higher morbidity, expressed by arrhythmogenic substrates, in CARD patients and either by the presence of subclinical alterations or arrhythmogenicity in IND patients. Understanding the role of this cytokine may provide useful information for better comprehension of the pathogenesis of ChD and contribute to the proper management and treatment of the patients.
